# Capgras Delusion in Posterior Cortical Atrophy–A Quantitative Multimodal Imaging Single Case Study

**DOI:** 10.3389/fnagi.2020.00133

**Published:** 2020-05-29

**Authors:** Matthias L. Schroeter, Franziska Albrecht, Tommaso Ballarini, Dominique Leuthold, Angela Legler, Simone Hartwig, Solveig Tiepolt, Arno Villringer

**Affiliations:** ^1^Max Planck Institute for Human Cognitive and Brain Sciences, Leipzig, Germany; ^2^Clinic for Cognitive Neurology, University of Leipzig, Leipzig, Germany; ^3^Department of Nuclear Medicine, University of Leipzig, Leipzig, Germany

**Keywords:** Balint’s syndrome, Capgras delusion, delusional misidentification syndrome, MRI, posterior cortical atrophy

## Abstract

Although Alzheimer’s disease presents homogeneous histopathology, it causes several clinical phenotypes depending on brain regions involved. Beside the most abundant memory variant, several atypical variants exist. Among them posterior cortical atrophy (PCA) is associated with severe visuospatial/visuoperceptual deficits in the absence of significant primary ocular disease. Here, we report for the first time a case of Capgras delusion—a delusional misidentification syndrome, where patients think that familiar persons are replaced by identical “doubles” or an impostor—in a patient with PCA. The 57-year-old female patient was diagnosed with PCA and developed Capgras delusion 8 years after first symptoms. The patient did not recognize her husband, misidentified him as a stranger, and perceived him as a threat. Such misidentifications did not happen for other persons. Events could be interrupted by reassuring the husband’s identity by the patient’s female friend or children. We applied in-depth multimodal neuroimaging phenotyping and used single-subject voxel-based morphometry to identify atrophy changes specifically related to the development of the Capgras delusion. The latter, based on structural T1 magnetic resonance imaging, revealed progressive gray matter volume decline in occipital and temporoparietal areas, involving more the right than the left hemisphere, especially at the beginning. Correspondingly, the right fusiform gyrus was already affected by atrophy at baseline, whereas the left fusiform gyrus became involved in the further disease course. At baseline, glucose hypometabolism as measured by positron emission tomography (PET) with F18-fluorodesoxyglucose (FDG-PET) was evident in the parietooccipital cortex, more pronounced right-sided, and in the right frontotemporal cortex. Amyloid accumulation as assessed by PET with F18-florbetaben was found in the gray matter of the neocortex indicating underlying Alzheimer’s disease. Appearance of the Capgras delusion was related to atrophy in the right posterior cingulate gyrus/precuneus, as well as right middle frontal gyrus/frontal eye field, supporting right frontal areas as particularly relevant for Capgras delusion. Atrophy in these regions respectively might affect the default mode and dorsal attention networks as shown by meta-analytical co-activation and resting state functional connectivity analyses. This case elucidates the brain-behavior relationship in PCA and Capgras delusion.

## Background

Alzheimer’s disease constitutes a major public health problem. Whereas its histopathology is rather homogeneous with amyloid and tau accumulation in the brain, it causes several clinical phenotypes (Warren et al., 2012). Clinically, the most frequent related dementia syndrome, typical Alzheimer’s dementia, is characterized by memory dysfunction (Dubois et al., 2007, 2014; McKhann et al., 2011). Beside the memory variant several atypical variants exist. Among them posterior cortical atrophy (PCA) is associated with severe visuospatial and visuoperceptual deficits in the absence of significant primary ocular disease (Warren et al., 2012; Crutch et al., 2017). Further PCA symptoms include simultanagnosia with or without optic ataxia or ocular apraxia, constructional dyspraxia, visual field defects, environmental disorientation, and Gerstmann’s syndrome. Remarkably, PCA is frequently diagnosed late in the course of the disease as the syndrome does not show apparent primary ocular disease giving easy explanation of problems and is a rare orphan disease, unfamiliar to most physicians.

Recently, delusional misidentification syndromes have been described in dementia syndromes, in particular in Alzheimer’s disease, dementia with Lewy bodies, and semantic dementia (Cipriani et al., 2013). These syndromes are psychopathologic phenomena, where patients consistently misidentify persons, places, objects, or events. In the Capgras delusion or syndrome of doubles, persons think that familiar persons are replaced by identical doubles or an impostor. Of note, there are no significant changes in the physical appearance of the misidentified objects. This syndrome was firstly described in 1923 by Capgras and Reboul-Lachaux, who coined it *illusion de sosies*. Capgras delusion is most often related to the spouse and siblings, i.e., people with whom the patient has strong emotional bonds and associative memories, where the double is assumed to have evil intent (Cipriani et al., 2013). Capgras delusion has been initially described in psychiatric diseases such as paranoid schizophrenia and schizoaffective disorder, later on in neurological diseases such as cerebrovascular disease, epilepsy, head injury, and dementia syndromes. Recent studies identified right frontal areas (Cipriani et al., 2013; Fiacconi et al., 2014; Darby and Prasad, 2016; Darby et al., 2017), right temporal/occipital cortex (Halligan and David, 2001; Metin et al., 2019), and retrosplenial cortex (Darby et al., 2017) as relevant for Capgras delusion. Here, we report the first case of Capgras delusion in a patient with PCA and identify its neural correlates.

## Case Presentation

The 57-year-old female patient presented together with her spouse to the Clinic for Cognitive Neurology at the University Hospital Leipzig, Germany, with visual impairment that had developed gradually over the last 3 years. The patient worked as a secretary. There, she recognized problems writing with ten fingers on the computer keyboard, using computer programs, organizing dates. Moreover, she and her husband described problems in everyday life, in particular losing the ability to identify correct time on watches, keep lines when reading and writing, open the door with a key, put a plug to a socket, switch on/off the kitchen stove and other kitchen equipment, put cutlery into the drawer, load the dishwasher, put shoes or clothing into cupboards, dressing, and word finding difficulties. The patient described visuospatial and visuoperceptual problems as “things don’t fit to each other,” “I cannot find any order.” Remarkably, three ophthalmologic investigations could not identify any specific related pathology, but recommended using glasses. One year before admission, a burn-out syndrome was diagnosed and the antidepressive drug venlafaxine administered.

### Investigations

Investigations with structural magnetic resonance imaging (MRI) 2 and 1 year before admission and visual evoked potentials (VEP) did not reveal any pathological findings. A lumbar puncture revealed reduced amyloid-beta 1–42 levels and normal values for total and phospho tau as well as negative protein 14-3-3 in cerebrospinal fluid (CSF).

#### Baseline Investigation

**FIGURE 1:**
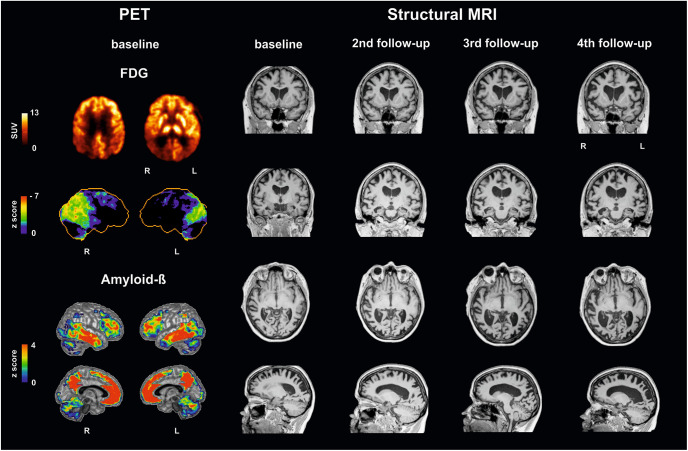
Multimodal imaging findings in a patient with posterior cortical atrophy at baseline and follow-up investigations. (Left) Positron emission tomography (PET) with F18-fluorodesoxyglucose (FDG-PET) reveals decreases in glucose metabolism: upper row in the patient (SUV standardized uptake value), second row compared to a healthy control cohort using NEUROSTAT/3D-SSP software (https://neurostat.neuro.utah.edu). Bottom rows show amyloid-beta accumulation in patient’s individual F18-florbetaben PET scan compared to a healthy control cohort using Hermes BRASS software (Hermes Medical Solution, Stockholm, Sweden). (Right) Structural magnetic resonance imaging (MRI), T1 sequence, illustrating atrophy. Note that Capgras delusion was firstly evident between third and fourth follow-up. L, left; R, right.

Figure 1 illustrates brain imaging findings. A structural MRI revealed brain atrophy especially in parietooccipital regions and, accordingly, enlargement of posterior ventricles. Single small white matter lesions did not reach pathologically relevant extent. Positron emission tomography (PET) with F18-fluorodesoxyglucose (FDG-PET) as a neuronal injury marker revealed glucose hypometabolism bilaterally in parietooccipital cortex, more pronounced on the right side, and in the right frontotemporal cortex. PET with F18-florbetaben showed amyloid accumulation in the gray matter of the neocortex (posterior cingulate, parietooccipital, and frontal cortex).

Clinical in-depth phenotyping revealed severe Balint’s syndrome with simultanagnosia, optic ataxia, ocular apraxia and visuospatial/visuoperceptual deficits, incomplete homonymous anopsia in the left lower quadrant, hemineglect on the left side, form and object agnosia. Although the patient did not recognize therapists in clinical routine that might indicate prosopagnosia, face recognition was possible, but with a reduced speed, in a facial recognition test for unknown faces. Deficits led to an impairment of exploration and reading abilities with problems in orientation in traffic, rooms, and on pages. Related clinical symptoms included alexia, agraphia, acalculia, deficits in attention, and with a lesser extent in memory and executive functions. The patient reached 24 out of 30 possible points in the Mini Mental State Examination (MMSE) 2 months before baseline investigation, and then she scored 23 points. The patient did not show any hints for proper aphasia. The patient was impaired in everyday life activities. Supplementary Video S1 illustrates clinical symptoms including simultanagnosia during visual exploration and object agnosia in clinical examination, optic ataxia combined with visuospatial and visuoperceptual deficits with problems in placing a plate into the dishwasher, putting a plug to a socket, and using a water boiler, and finally tactile compensation of deficits. Although the patient had problems in recognizing a fork or spoon visually (see third movie part for object agnosia), she could easily identify these items based on tactile information without visual information (eyes had been covered by blindfold, see last movie part for tactile compensation). Interestingly, this strategy was also applied successfully by the patient spontaneously when she failed in her first attempt with putting a plug to a socket (see second movie part for optic ataxia).

#### Differential Diagnosis

Differential diagnosis of neurodegenerative diseases has to take clinical syndromes and biomarkers from imaging and CSF as surrogate indicators for histopathology into account as recently suggested in clinical diagnostic criteria (Schroeter et al., 2009; McKhann et al., 2011; Schroeter and Neumann, 2011). In accordance with this conceptual shift, Crutch et al. (2017) suggested a three-level classification framework for PCA comprising both syndrome and disease-level descriptions. On *classification level 1* the patient fulfilled the core features of the PCA clinico-radiological syndrome, i.e., clinical criteria with insidious onset, gradual progression, prominent early disturbance of visual and/or other posterior cognitive functions, and the cognitive features with space perception deficit, simultanagnosia, object perception deficit, constructional dyspraxia, environmental agnosia, oculomotor apraxia, dressing apraxia, optic ataxia, alexia, acalculia, agraphia, and homonymous visual field defect. Later on (see below), further discussed PCA features, apperceptive prosopagnosia, finger agnosia, and left/right disorientation were also fulfilled (Gerstmann’s syndrome). Neuroimaging indicated predominant occipitoparietal or occipitotemporal atrophy on MRI and parietooccipital hypometabolism on FDG-PET. *Classification level 2* revealed pure PCA without fulfilling core clinical criteria for any other neurodegenerative syndrome. *Classification level 3* provided biomarker-evidence for Alzheimer’s disease as the underlying pathology in accordance with the suggested criteria for this disease, i.e., decreased amyloid beta 1–42 in CSF and neocortical amyloid-beta accumulation in PET (Dubois et al., 2007, 2014). There was no evidence for familial/genetic etiology and no autopsy information available. As suggested in McKhann et al. (2011) there was evidence for both Alzheimer’s disease etiology (amyloid alterations as shown in PET and CSF) and neuronal injury (FDG-PET, structural MRI; for imaging meta-analyses see Schroeter and Neumann, 2011, and Schroeter et al., 2009). In sum, the patient suffered from PCA on a syndrome-clinical level with evidence for underlying Alzheimer’s disease on imaging and biomarker levels.

### Treatment

An acetylcholine-esterase-inhibitor (galantamine) starting with a daily dose of 8 mg was administered due to underlying Alzheimer’s disease. Deficits were treated in a day clinic out-patient setting with a multimodal approach including orthoptists, occupational therapists, neuropsychologists, patholinguists, and physiotherapists. Support by occupational therapy, patholinguistic therapy, and psychotherapy was recommended to be continued after discharge.

### Outcome

#### First Follow-Up Investigation

Eight months later the patient was re-examined. Dosage of galantamine was increased to 24 mg daily in the meantime. Intensive therapy by orthoptist and patholinguist improved reading of words and sentences, whereas reading of numbers and writing of words and sentences did show more mistakes. These changes were related to a decline in visuospatial and visuoperceptual functions, the left-sided hemineglect, and simultanagnosia. Whereas at first investigation the patient could perceive three objects simultaneously, at follow-up only two objects were detected. The patient was functionally blind and had to rely on support by her spouse. Prosopagnosia was detected in facial recognition test and prosopagnosia test. MMSE score reached now 22 out of 30 possible points.

#### Second Follow-Up Investigation

During the follow-up 17 months later, the patient reported further deterioration of visuospatial and visuoperceptual abilities including reading and writing, whereas she could better compensate deficits by putting things into cupboards by using strategies. She also told that she identified persons now by their voice and/or gait instead of recognizing their face. She reported failing sitting on chairs by sitting beside it. The husband reported depressive mood. Coxarthrosis required implantation of endoprostheses in both hip joints. In the meantime, dosage of galantamine was reduced to 16 mg daily due to nausea as a side effect. Investigations revealed additionally finger agnosia, deficits in body schema, and temporal orientation. The neuropsychiatric inventory excluded changes in personality/behavior besides slightly depressive mood. As illustrated in Figure 1 structural MRI indicated regional atrophy with slight increase in extent but without qualitative changes. Semantic abilities were now slightly impaired. Symptoms of the Balint’s syndrome, especially simultanagnosia, and cognitive abilities showed further decline if compared with earlier investigations (patient could perceive only one object now at one time, MMSE score 18 out of 30 points). These higher deficits led to misinterpretations of everyday usage objects, persons, and stronger impairments in everyday activities. Occupational therapy was now recommended in the patient’s household.

#### Third Follow-Up Investigation

At follow-up 17 months later, the patient and spouse reported further deterioration in higher order visual processing. According to the spouse the patient once had the illusion of people sitting in the garden, which were actually not there. This never happened again. Furthermore, the patient suspected that her husband would be interested in other women. Structural MRI revealed again severe cortical and subcortical atrophy, now with temporomesial atrophy (medial temporal lobe atrophy according to Scheltens’ 2 left/3 right; at baseline and at second follow-up 1 left/2 right). Thorough neuropsychological testing was not possible anymore due to deficits’ severity.

#### Fourth Follow-Up Investigation

The patient and spouse visited the clinic 14 months later. They reported further general decline of visuospatial/visuoperceptual abilities deteriorating everyday life activities. She could not use a knife and fork during eating appropriately. The husband added that the patient used her left hand less. Symptoms suspicious for hallucinations or paranoia were not reported any more. Most remarkably, the husband now stated that the patient sometimes did not recognize him and misidentified him rather as a stranger (Supplementary Video S2 taken by spouse). During these episodes she also perceived him as a threat; once or two times she even told that she had been raped. These events occurred now daily with a duration of 1 h. Such misidentifications would not happen for other persons. To interrupt such events by re-identifying the spouse as her husband he would have to call their children or the patient’s female friend by phone, to calm her and reassure her regarding the husband’s identity. Structural MRI (Figure 1) revealed more widespread atrophy compared with the previous MRIs, especially in temporoparietooccipital areas. Temporomesial atrophy reached now stage 3 bilaterally on the Scheltens’ scale. In the meantime, the frontal cortex was also involved. Ventricles were largely extended especially in posterior and temporal regions. Neuropsychological investigations, only possible for rough tests, showed further cognitive decline with now 4 out of 30 possible points in the MMSE. Visuospatial and visuoperceptual abilities, the Balint’s syndrome, and left-sided hemineglect were further deteriorated. Although low vision could be compensated by tactile information during previous examinations, this compensatory strategy was not applicable any more. Orientation was even impaired in their own flat. To cope with misidentification events the antipsychotic quetiapine and behavioral interventions were recommended beside respite care, occupational therapy at home, and support by assistants.

### Single-Subject Voxel-Based Morphometry

To validate the impact of structural imaging changes on the disease course and, in particular, the development of Capgras delusion quantitatively, we performed a single-subject voxel-based morphometry (VBM) analysis using MRI data. Here, progress of brain atrophy was quantitatively analyzed over time in the case-subject by comparing each timepoint of MRI scans to a healthy elderly cohort.

#### Method

Voxel-based morphometry identified differences that are specific for each timepoint in the patient’s disease course compared to a healthy control cohort. Here, 42 healthy controls (mean age ± SD = 69.6 ± 6.6 years) were contrasted to the four MRI measurements of the patient (baseline, age = 57.8; second follow-up, age = 60.0; third follow-up, age = 61.2; fourth follow-up, age = 62.4 years) in four separate analyses.

Voxel-based morphometry was performed applying the CAT12 toolbox (University of Jena, Germany) with SPM12 (University College London, United Kingdom). Images were spatially normalized, segmented, modulated by the amount of linear and non-linear deformations, and smoothed with a Gaussian kernel of 8-mm full-width at half-maximum. Voxel-wise Student’s *t*-tests were performed to compare each timepoint of the patient with controls. Nuisance covariates were used to control for differences in age and total intracranial volume. Significant clusters were detected using an uncorrected voxel-threshold of *p* < 0.005 and a family-wise error (FWE) corrected cluster-threshold of *p* < 0.05. To visualize the disease progression, resulting gray matter volume (GMV) maps from the single timepoints comparisons were binarized and superimposed to create an overlay analysis.

As aforementioned, the Capgras delusions emerged between the last two visits. Accordingly, we regarded atrophy changes between the third and fourth follow-ups as the most informative, as they are timely coincident with the emergence of the symptoms of interest. In order to graphically display the trend over time in GMV reductions, we performed an additional data-driven region-of-interest (ROI) analysis. Using marsbar v.0.44^1^, we extracted the average GMV values from data-driven ROIs defined according to the atrophy pattern characteristic of the case-patient during the fourth follow-up. Namely, we created three ROIs, one encompassing the overall PCA pattern (i.e., including extensively occipital and temporoparietal cortex) and two from the brain regions that emerged concurrently with the Capgras delusion in the transition between the third and fourth follow-up, i.e., posterior cingulate gyrus/precuneus and middle frontal gyrus/frontal eye field (respectively, centered on the MNI x, y, z coordinate 4, −32, 44 and 28, 0, 51). GMV values for the healthy controls were summarized in a boxplot and values for each timepoint for the patient are visualized separately. In addition, we also extracted the total GMV of the patient at each timepoint to measure overall atrophy changes.

#### Results

**FIGURE 2:**
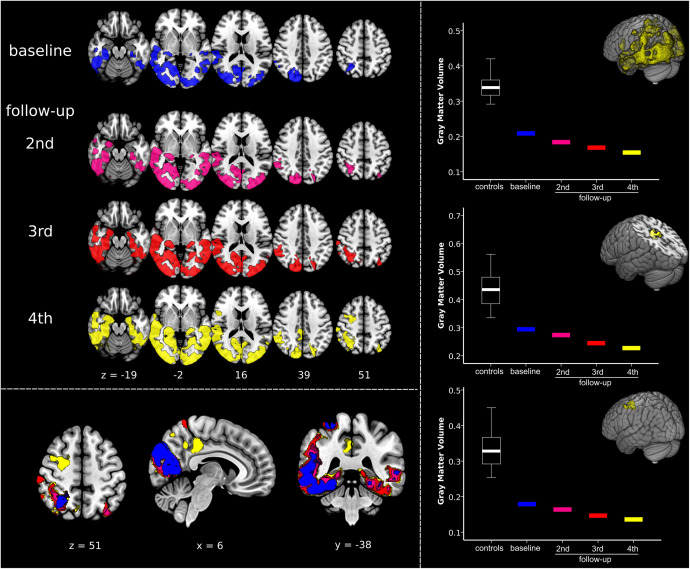
Quantifying dynamic structural imaging changes in single-subject voxel-based morphometry analysis. (Left) Top panel displays results of the voxel-based morphometry analysis showing, at each timepoint, the impairment in gray matter volume (GMV) in the patient compared to a normative elderly healthy control group. Bottom panel shows overlay of patient’s atrophy across the four timepoints, clearly displaying disease progression over time. The same results are presented in Supplementary Video S3. (Right) Results of the regions-of-interest (ROI) analysis illustrating atrophy progression (GMV decline). The upper graph is related to the progression of the disease in the overall disease-specific pattern, i.e., regions affected at fourth follow-up. The middle and the bottom graphs show GMV changes in the clusters timely associated with the emergence of Capgras delusion, i.e., right posterior cingulate gyrus/precuneus, and middle frontal gyrus/frontal eye field (respectively, centered at MNI coordinates x, y, z 4, –32, 44 and 28, 0, 51). Significant results are shown at *p* < 0.05 family-wise error corrected (FWE) at cluster level (cluster forming threshold *p* < 0.005). Images are displayed in radiological convention: left of the figure displayed on the right of the image.

As illustrated in Figure 2 and Supplementary Video S3 the single-subject VBM analysis revealed general progressive GMV decline in occipital and temporoparietal areas from baseline to fourth follow-up. Generally, atrophy involved more the right than the left hemisphere, especially in the beginning of imaging investigations. Atrophy was already observed in the right fusiform gyrus at baseline, whereas the left fusiform gyrus appeared relatively spared at baseline but was severely compromised at fourth follow-up. Most specific changes during transition from the third to the fourth follow-up, i.e., the appearance of the Capgras delusion, are illustrated in yellow color in the left bottom panel in Figure 2 and in Supplementary Video S3. New GMV reduction was found at this timepoint in the posterior cingulate gyrus/precuneus, as well as in the right middle frontal gyrus/frontal eye field. The ROI analyses revealed that summarized GMV values in the healthy control group were always higher in all of the three investigated regions. GMV values declined with each follow-up in all of the three ROIs (Figure 2, right panel). Of note, no sharp decline in GMV was detected in any of the three ROIs, rather a slow, progressive decrease. A similar decrease rate was also evident in the overall progression of atrophy as quantified by a gradual loss of total GMV from baseline to the last follow-up (468 cm^3^ 450 cm^3^ 417 cm^3^ 403 cm^3^).

### Extracting Functionally Impaired Brain Networks by Connectivity Analyses

#### Method

To further explore the significance of atrophy differences between the third and the fourth follow-up for functionally associated brain networks, we applied two additional analytical techniques via Neurosynth^2^ (Yarkoni et al., 2011; see also Schroeter et al., 2020): (1) meta-analytic co-activation modeling (MACM), which identifies co-activation of brain regions across studies reported in the Neurosynth database (*N* = 14,371 as of March 2020); and (2) seed-based resting state functional connectivity based on a sample of 1,000 healthy controls (Choi et al., 2012). The seeds for both analyses were created as 6 mm radius spheres centered in the atrophy maxima of the two clusters that differentiated the patients and controls at the fourth follow-up but not at previous timepoints, i.e., in the posterior cingulate/precuneus and in the frontal gyrus/frontal eye fields (coordinates in MNI space 4, −32, 44, and 28, 0, 52).

#### Results

**FIGURE 3:**
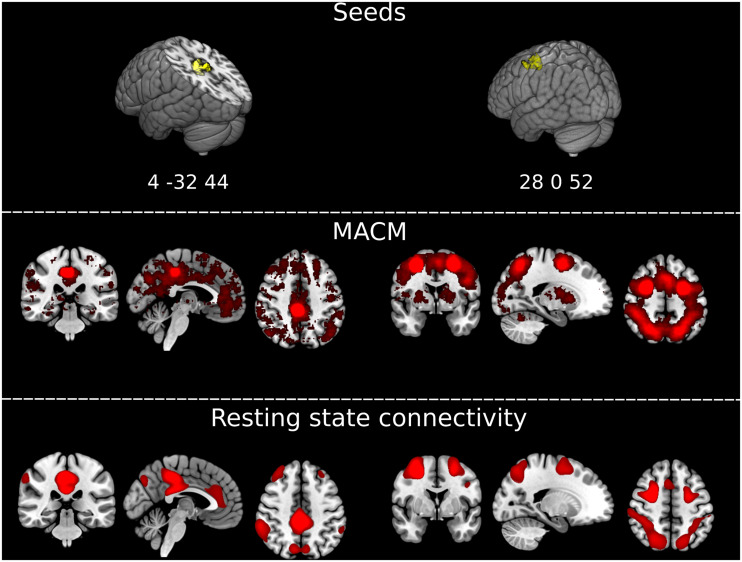
Extracting functionally impaired brain networks by mapping atrophy to resting-state networks and functional co-activations. (Top) Graphical representation of the clusters of brain atrophy timely associated with the emergence of Capgras delusion, i.e., right posterior cingulate gyrus/precuneus, and middle frontal gyrus/frontal eye field. Coordinates of the maxima for each of the two clusters are reported in MNI space in the figure: x, y, z 4, –32, 44, and 28, 0, 52. (Middle) Results of the meta-analytic co-activation modeling (MACM) across >14,000 fMRI studies from the Neurosynth database (https://neurosynth.org/). Red blobs represent *z*-scores after correction for false discovery rate (FDR). (Bottom) Results of the seed-based resting-state functional connectivity analysis on an independent sample of *N* = 1,000 healthy controls generated with Neurosynth. Red blobs show Pearson’s correlations with the seed, thresholded at *r* ≥ 0.2, uncorrected. Images are displayed in radiological convention. Left of the figure displayed on the right of the image.

Results from both MACM and seed-based resting-state functional connectivity were highly consistent for each seed (Figure 3). The seed in the right frontal gyrus/frontal eye field showed preferential co-activations and functional connectivity with its contralateral homolog, and with the bilateral superior parietal cortex, closely resembling the architecture of the dorsal attention network. The seed in the posterior cingulum/precuneus showed to be functionally connected/co-activated with the bilateral inferior parietal cortex and medial frontal and anterior cingulate cortex, well-known components of the default mode network. The two brain networks connected/co-activated with the seeds were topographically dissociated.

## Discussion

Here, we report, to the best of our knowledge, the first case of Capgras delusion in a patient with PCA. PCA was diagnosed in this female patient 3 years after beginning of clinical disturbances starting at 54 years of age. Clinical in-depth phenotyping and investigations with multimodal imaging and biomarkers from CSF revealed pure PCA fulfilling diagnostic criteria in the three-level classification framework for PCA as suggested by Crutch et al. (2017) comprising both syndrome- and disease-level descriptions. Beside the full clinical syndrome, the patient showed typical findings in neuroimaging with MRI and FDG-PET. Amyloid-beta PET and amyloid-beta in CSF provided biomarker-evidence for Alzheimer’s disease as the underlying pathology (Dubois et al., 2007, 2014; Warren et al., 2012).

Most importantly, approximately 5 years after first diagnosis of PCA and subsequent stepwise decline in higher order visual processing and cognitive abilities, the patient developed a delusional misidentification syndrome—Capgras delusion—which has never been described in the literature in PCA before. Harciarek and Kertesz (2008) investigated the prevalence of delusional misidentification syndromes in a large cohort with neurodegenerative diseases. Generally relatively high prevalence was observed in Alzheimer’s disease (15.8%) and dementia with Lewy bodies (16.6%), lower values in semantic dementia (8.3%). A recent case study also reported Capgras delusion in a patient with anterior left temporal lobe atrophy showing clinically symptoms of semantic dementia and behavioral variant frontotemporal dementia (Metin et al., 2019).

Capgras delusion is relatively common in Alzheimer’s disease (Cipriani et al., 2013). Surrogate biomarkers for histopathology revealed Alzheimer’s disease in our patient as well. However, the patient never showed hints for typical Alzheimer’s dementia with prominent memory impairment or Lewy body dementia beside one appearing non-recurring visual hallucination. Capgras delusion appeared in our patient with an age of 62 years, earlier than described in Alzheimer’s disease with a mean age of 72 to 73 years (Cipriani et al., 2013). Because in our patient Capgras delusion developed in the course of the disease 8 years after clinical onset of PCA, one might assume that (histopathologically) underlying Alzheimer’s disease might also be the cause for this syndrome in our patient.

Cipriani et al. (2013) discuss that patients with Capgras delusion are characterized by unilateral brain lesions in the right hemisphere, in particular frontal areas (see also Darby and Prasad, 2016), and that underactivity in the perirhinal cortex seems to lead to loss of familiarity in this syndrome. Fiacconi et al. (2014) showed that medial prefrontal cortex atrophy was associated with Capgras delusion in dementia with Lewy bodies. Other authors (Halligan and David, 2001) suggest that Capgras delusion is related to alterations in information processing in the right temporal/occipital cortex. They propose that delusion arises from a deficit in face processing, where information about a subject’s facial identity is processed in brain regions disconnected from relevant affective information regarding the full identity of the face’s owner. The delusion would occur if the patient aims at explaining or reconciling the subject’s identity without having its affective information. Note that delusional misidentification syndromes have also been observed in semantic dementia (Harciarek and Kertesz, 2008) well known to be related to anterior temporal hypometabolism and atrophy (Schroeter et al., 2007; Bisenius et al., 2016, 2017). Capgras delusion has also been reported in a patient with anterior temporal atrophy showing symptoms of behavioral variant frontotemporal dementia besides that of semantic dementia (Metin et al., 2019).

Imaging findings in our patients support these assumptions (see Figure 1). FDG-PET, as a neuronal injury marker, revealed glucose hypometabolism in the right frontotemporal cortex, and parietooccipital cortex (the latter known for PCA; Warren et al., 2012) more pronounced right-sided, already at baseline. As illustrated in Figure 1, atrophy as measured with structural MRI spread during the course of the disease, involving at last follow-up investigation also frontal areas beside occipitoparietotemporal areas including the fusiform gyrus/parahippocampal and perirhinal cortex pronounced on the right side, where early atrophy mainly in occipitoparietal areas is described in PCA (Warren et al., 2012; Firth et al., 2019).

The in-depth single-subject VBM analysis based on structural MRI quantified the atrophy changes across the disease course (Figure 2). It showed general progressive GMV decline in occipital and temporoparietal areas, remarkably involving more the right than the left hemisphere, especially at the beginning. Correspondingly, the right fusiform gyrus was already affected by atrophy at baseline, whereas the left fusiform gyrus became involved in the further disease course. Most remarkably, the transition analysis from the third to the fourth follow-up revealed new brain atrophy in the right posterior cingulate gyrus/precuneus, as well as in the right middle frontal gyrus/frontal eye field functionally connected to the default mode and dorsal attention networks that might have led to the development of the Capgras delusion in this patient. This single-subject VBM analysis elucidates the brain-behavior relationship in Capgras delusion in PCA and extracts these brain regions as particularly relevant for Capgras delusion. Note that our results agree also with histopathological studies reporting neurofibrillary tangles in occipital lobe, temporoparietal junction, and posterior cingulate in PCA, whereas amyloid plaques are more variable (Warren et al., 2012).

Our results confirm right frontal areas as relevant for Capgras delusion as suggested by Cipriani et al. (2013) and Darby and Prasad (2016), although one has to mention here that the right temporal/occipital cortex had already been affected by atrophy (and hypometabolism) in this patient at the beginning in correspondence with Halligan’s and David’s (2001) framework. Moreover, the extent of the identified new brain regions associated with the Capgras delusion is also influenced by the chosen statistical threshold as the ROI analyses rather show a stepwise decline in GMV there.

The results of our transition analysis from the third to the fourth follow-up, which identified the posterior cingulate gyrus/precuneus and the right middle frontal gyrus/frontal eye field as relevant for Capgras delusion, are also in agreement with a recent study by Darby et al. (2017). The authors applied lesion network mapping to identify common hubs in the connectome related to Capgras and other delusional misidentification syndromes in stroke patients. Brain lesions were functionally connected to the retrosplenial cortex (as well as posterior cingulate gyrus/precuneus, see their Figure 3), a region most activated in functional MRI studies of familiarity, and to the right frontal cortex, in particular the ventral frontal cortex/anterior insula, regions most activated in functional MRI studies of expectation violation, a component of belief evaluation. The authors suggest that impaired familiarity perception and belief evaluation might lead to delusional misidentifications. As our patient study identified the same regions, one might hypothesize analog mechanisms here. Note that the transition analysis from the third to the fourth follow-up additionally indicated atrophy in the right insula in our patient as illustrated in Supplementary Video S3.

One might object that severe visuo-perceptual deficits including prosopagnosia (the latter confirmed during first follow-up investigation in the patient) and leading to functional blindness might generally hamper person perception in PCA as the disease generally impairs perception of the misidentified object. Radicalizing this argument one even might hypothesize that true Capgras phenomena generally cannot occur in PCA and the syndrome’s observation is an artifact in this patient. However, several points support our assumption that the patient experienced a real Capgras syndrome. (i) The patient described compensatory strategies, i.e., identifying persons by their voice and/or gait instead of recognizing their face. Hence, person perception was generally possible in the patient, although not via face perception. (ii) This hypothesis is further supported by the fact that person perception was selectively impaired only for the spouse in our patient and even occurred only intermittently. Although the patient had not shown any hints for epileptic seizures until fourth follow-up, such seizures developed 4 months thereafter. The antiepileptic drug valproate was administered beside antipsychotics after two other seizures (6 and 9 months after fourth follow-up). Subsequently, Capgras delusion disappeared either due to progress of the disease (MMSE 2/30 points 8 months after fourth follow-up), antipsychotics, or antiepileptics. (iii) The specificity of the Capgras delusion in PCA is also supported by other case studies reporting Capgras delusion in a patient with blindness (referred to in Cipriani et al., 2013). Cipriani et al. (2013) conclude that intact vision seems not to be necessary for Capgras delusion, and that also other senses and their misinterpretation, such as hearing and touch, might contribute to this phenomenon. However, the high prevalence of visuoperceptual and visuospatial deficits in PCA patients might have kept other authors from interpreting misidentifications of relatives, etc., as Capgras phenomena.

For choline esterase inhibitors effects on functional and structural imaging have been reported in Alzheimer’s disease and its prestage mild cognitive impairment (Prins et al., 2014; Kim et al., 2017). Because the patient was treated with galantamine, we cannot rule out modulating effects on our results, although one would expect here rather a deceleration of atrophy/hypometabolism rates.

One might further discuss how atrophy in frontal areas can be related to Capgras delusion in this patient as many previous studies have shown significant atrophy in PCA patients also in frontal areas when compared to healthy controls. Indeed, frontal areas might be involved in PCA, but in late stages only. Recently, Firth et al. (2019) investigated atrophy longitudinally in PCA in a large cohort with approximately 100 patients. Patients with PCA showed early occipitoparietal atrophy, with subsequent higher rates of temporal atrophy, whereas atrophy in frontal areas appeared much later and to smaller extent. This finding agrees with results in our patient showing late and regionally restricted frontal atrophy. Still, the question remains why atrophy in the (frontal) brain regions might lead to Capgras delusion in this single patient but not in other patients with PCA. Here one has to state that it is methodologically problematic to compare group studies with single case studies. Group studies conduct group statistics across numerous subjects, accordingly statistical power is large, clusters/effects are large. But one cannot conclude anything about single subjects. Moreover, individual clinical deficits are not taken into account beside individual atrophy patterns. One might conclude that group data and single subject analyses are in principle not comparable. Our patient supports evidence—in a lesion model based on longitudinal single-subject VBM analysis—that PCA affects specific structural and functional neural networks leading to Capgras delusion.

## Patient’s Perspective

Due to the severity of impairment the patient could not comment herself. The spouse reported that he was surprised and terrified of the speed of the deficits’ development.

## Data Availability Statement

Data for this article are not publicly available to guarantee patient’s confidentiality. Data are available on reasonable request.

## Ethics Statement

The patient took part in the multi-centric German Consortium for Frontotemporal Lobar Degeneration study 3 (Otto et al., 2011). The study was authorized by the local ethics committees of the participating centers, in line with the Declaration of Helsinki (Ethic’s Committee University of Leipzig ID137-11-18042011 – Ethik-Kommission an der Medizinischen Fakultät der Universität Leipzig, Karl-Sudhoff-Institut für Geschichte der Medizin und der Naturwissenschaften, Käthe-Kollwitz-Straße 82, 04109 Leipzig). The patient gave written informed consent for participation Furthermore, the spouse, as the patient’s legal representative, gave informed consent for publication as a case report. A copy of the written consent is available for review to the Editor of this journal.

## Author Contributions

MS, DL, AL, and SH investigated and treated the patient. ST measured and analyzed PET imaging data. FA and TB analyzed MRI data quantitatively. MS wrote the first version of the manuscript. All authors commented, rewrote, and finally proved the manuscript.

## Conflict of Interest

The authors declare that the research was conducted in the absence of any commercial or financial relationships that could be construed as a potential conflict of interest.

## Footnotes

1 http://marsbar.sourceforge.net

2 http://www.neurosynth.org

## Supplementary Material

VIDEO S1 Clinical symptoms as shown by the patient, i.e., simultanagnosia, optic ataxia, object agnosia, and tactile compensation of deficits.

VIDEO S2 Capgras delusion as shown by the patient in contact with spouse.

VIDEO S3 Atrophy in patient’s brain as revealed by single-subject voxel-based morphometry compared to a normative elderly healthy control group. Movie shows overlay of patient’s atrophy across the four timepoints, clearly displaying disease progression over time. For color coding see legend to Figure 2.
